# Common Avian Infection Plagued the Tyrant Dinosaurs

**DOI:** 10.1371/journal.pone.0007288

**Published:** 2009-09-30

**Authors:** Ewan D. S. Wolff, Steven W. Salisbury, John R. Horner, David J. Varricchio

**Affiliations:** 1 Department of Pathobiological Sciences, School of Veterinary Medicine, University of Wisconsin, Madison, Wisconsin, United States of America; 2 School of Biological Sciences, The University of Queensland, Brisbane, Queensland, Australia; 3 Vertebrate Paleontology, Carnegie Museum of Natural History, Pittsburgh, Pennsylvania, United States of America; 4 Museum of the Rockies, Montana State University, Bozeman, Montana, United States of America; 5 Department of Earth Sciences, Montana State University, Bozeman, Montana, United States of America; Stanford University, United States of America

## Abstract

**Background:**

*Tyrannosaurus rex* and other tyrannosaurid fossils often display multiple, smooth-edged full-thickness erosive lesions on the mandible, either unilaterally or bilaterally. The cause of these lesions in the *Tyrannosaurus rex* specimen FMNH PR2081 (known informally by the name ‘Sue’) has previously been attributed to actinomycosis, a bacterial bone infection, or bite wounds from other tyrannosaurids.

**Methodology/Principal Findings:**

We conducted an extensive survey of tyrannosaurid specimens and identified ten individuals with full-thickness erosive lesions. These lesions were described, measured and photographed for comparison with one another. We also conducted an extensive survey of related archosaurs for similar lesions. We show here that these lesions are consistent with those caused by an avian parasitic infection called trichomonosis, which causes similar abnormalities on the mandible of modern birds, in particular raptors.

**Conclusions/Significance:**

This finding represents the first evidence for the ancient evolutionary origin of an avian transmissible disease in non-avian theropod dinosaurs. It also provides a valuable insight into the palaeobiology of these now extinct animals. Based on the frequency with which these lesions occur, we hypothesize that tyrannosaurids were commonly infected by a *Trichomonas gallinae*-like protozoan. For tyrannosaurid populations, the only non-avian dinosaur group that show trichomonosis-type lesions, it is likely that the disease became endemic and spread as a result of antagonistic intraspecific behavior, consumption of prey infected by a *Trichomonas gallinae*-like protozoan and possibly even cannibalism. The severity of trichomonosis-related lesions in specimens such as *Tyrannosaurus rex* FMNH PR2081 and *Tyrannosaurus rex* MOR 980, strongly suggests that these animals died as a direct result of this disease, mostly likely through starvation.

## Introduction

Dinosaur fossils often display evidence of injuries that can be attributed to accidents, old age or metabolic disorders. Among predatory dinosaurs, the most commonly encountered abnormalities are bite-marks and related bone traumas on the head [1], [2]. Tooth strike trauma in theropods occurs on or around the face, particularly on the maxillary rostrum and dentary bones, and the injuries are often undergoing active healing at the time of death. The associated lesions include solitary or multiple round or oval-shaped puncture marks, and elongate gouges and scores caused by tooth tips being dragged across the surface of the bone. Depending on the extent to which they have healed, puncture marks typically have centrally infolded margins, whereas gouges have ragged margins [1]. Comparisons with unhealed tooth marks on prey suggests that wounds of this type were inflicted by other large theropods, most likely conspecifics, as a result of territorial disputes, mating and possibly cannibalism [1], [2]. Although the possibility remains that theropods within the same geographic range [e.g. *Daspletosaurus* and *Albertosaurus*] could have made similar bite marks on each other's skulls, such interaction amongst relatively few individuals is unlikely. Furthermore, bite marks made by *Tyrannosaurus rex* are unmistakable and no similarly sized predator is known from the latest Maastrichtian of North America.

**Figure 1:**
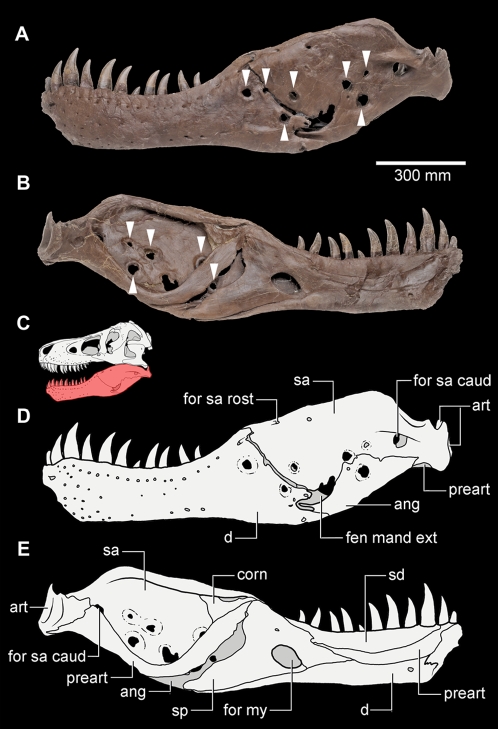
*Tyrannosaurus rex* (FMNH PR2081). Left mandibular ramus exhibiting multiple trichomonosis-type lesions (indicated by arrows); (A and D), lateral view (photo; schematic interpretation). (B and E) medial view (photo; schematic interpretation). (C), schematic interpretation of the reconstructed skull of FMNH PR2081 in left lateral view with the mandibular ramus shown in red. Anatomical abbreviations: ang, angular; art, articular; corn, coronoid; d, dentary; fen mand ext, external mandibular fenestra; for my, mylohyal foramen; for sa caud, caudal surangular foramen; for sa rost, rostral surangular foramen; preart, prearticular; sa, surangular; sd, supradentary; sp, splenial. a and b modified from ©1999 The Field Museum, GEO86260_7c and GEO86262_4c, respectively. Photographer John Weinstein.

Many tyrannosaurids display a second category of cranial abnormalities distinct from the traumas associated with head-biting. These abnormalities are smooth-edged fully erosive lesions that are most commonly present in the mandible. They occur either singly or in multiples and vary in size from millimeters to several centimeters in diameter. The edges of the bone in these lesions varies from tapered to sub-rounded, but all exhibit extensive surrounding remodelling of the bone surface texture, occasionally accompanied by mild rugosity of the bone surface, as is seen in FMNH PR2081 [3, Supporting Information S1]. These smooth-edged lesions have had only anecdotal mention previously, and their origin is poorly understood. For example, cylindrical erosive lesions on the surangular and dentary of ‘Sue’ (FMNH PR2081; Fig. 1) were attributed to an *Actinomyces bovis* infection [4]. While actinomycosis leads to extensive osteomyelitis in hominids and other mammals [5], bone-penetrating mandibular lesions from *Actinomyces bovis* infections in extant archosaurs remain unknown [6], [7], making this diagnosis unlikely.

To investigate the significance of these unusual smooth-edged abnormalities in tyrannosaurids, a detailed oral pathology survey of fossil and extant archosaurs was undertaken. Herein we demonstrate that these abnormalities have a strong resemblance to those lesions resulting from an infectious disease known to occur in modern birds called trichomonosis. This finding not only establishes that some non-avian theropods had a similar infectious disease to that found in living birds, but also suggests a similar or common immune response to the disease.

## Results

**Figure 2:**
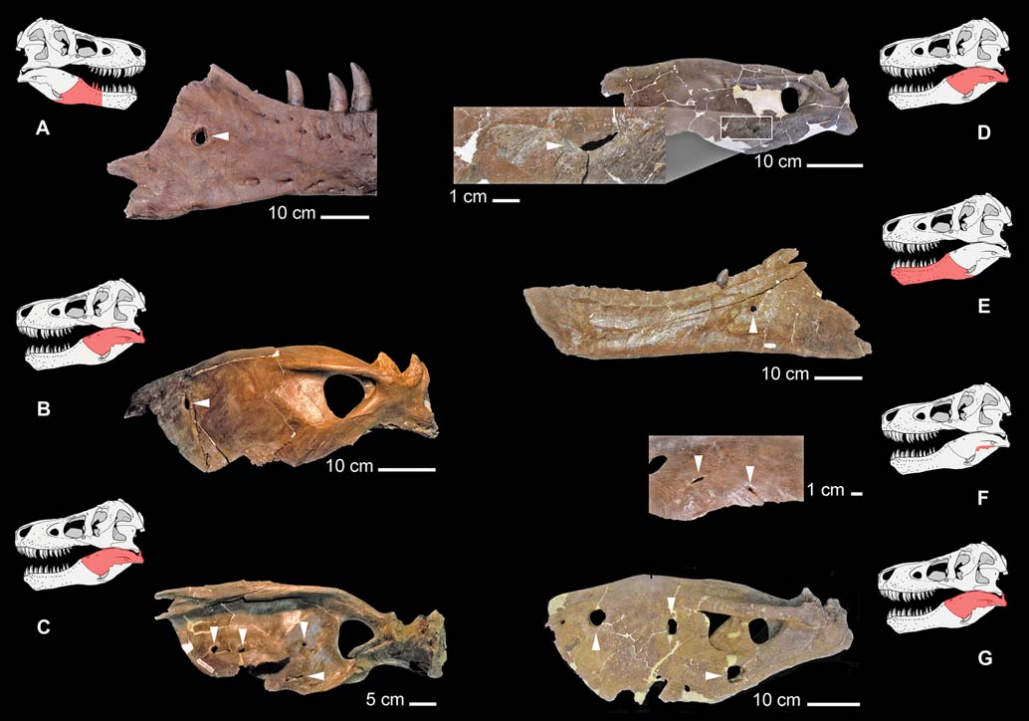
Tyrannosaurid mandibular pathology (arrows indicate trichomonosis-type lesions; the position of each specimen is shown in red on the accompanying schematic interpretation of the reconstructed skull of *Tyrannosaurus rex*, FMNH PR2081); (A), *Tyrannosaurus rex* (holotype; CMNH 9380) right caudal mandibular ramus in lateral view, displaying a large circumscribed erosive lesion on the caudal part of the dentary. (B) *Daspletosaurus torosus* (RTMP 2001.36.01) left caudal mandibular ramus in lateral view, displaying a single erosive lesion. (C), *Daspletosaurus torosus* (RTMP 2001.36.01) multiple erosive lesions are visible on the right surangular in medial view. (D), *Albertosaurus sarcophagus* (RTMP 1981.10.01), left caudal mandibular ramus in lateral view, showing a slit-shaped trichomonosis-type lesion in the middle of the angular, (enlarged area, inset). (E) *Tyrannosaurus rex* (MOR 1125), caudal part of left dentary in lateral view, showing a cylindrical-shaped lesion, with smooth edges and almost no surrounding bony alteration. (F), *Daspletosaurus torosus* (RTMP 94.143.1), surangular in ventral view, with one slit-shaped lesion and one intermediate lesion. The close-up shows the smooth edges and limited surrounding bony alteration characteristic of a trichomonosis-type lesion. (G), *Tyrannosaurus rex* (MOR 980), left surangular in lateral view, exhibiting multiple oval- to sub-oval-shaped lesions.

Nearly 15% of the 61 tyrannosaurid individuals examined during this study exhibited trichomonosis-type lesions on the mandible (Figure 1, Figure 2, and Supporting Information S1). In seven out of nine cases, the trichomonosis-type lesions showed a unilateral distribution (Figure 2, A, D, E, F and G, and Supporting Information S1). In the remaining cases, they occurred on both sides of the mandible (Figure 1, Figure 2, B and C, and Supporting Information S1). These abnormalities comprise multiple chronic smooth-edged erosive lesions with a focal distribution in the caudal part of the mandible, usually on the surangular and less commonly on the dentary (Figure 1, Figure 2, and Supporting Information S1). The abnormalities typically have a circular or slit-like shape, with moderate surrounding thickening of the bone. In the majority of cases, these lesions exhibit neither alteration to the bone fabric, deformation of the periosteal bone nor exposure of the endosteal bone. Instead, the outward appearance often consists of a developmental change in the bone with no indication of any obvious mechanism of formation.

The position and morphology of these lesions does not correspond with any fenestrae or foramina typically seen in non-avian dinosaurs, birds or other archosaurs, so individual variation in morphology can be ruled out [8]. The known pathology of modern archosaurs, on the other hand, provides useful insights into the nature of these abnormalities.

Superficial injuries in extant crocodylians usually heal, leaving scars on the skin [9]–[13]. Depending on the degree to which they heal, superficial wounds may form a point of entry for infectious agents such as ‘crocodile/caiman pox’ (*Parapoxvirus*) [14]. Crocodylian poxvirus is similar to a poxviral disease of modern birds of prey known as ‘raptor pox’, where crusty nodular lesions form within the skin [14]. However, lesions from these diseases are proportionately very small, and their morphology does not compare well with the trichomonosis-type lesions seen on tyrannosaurid mandibles.

Deeper facial wounds in extant crocodylians, particularly those that penetrate bone, often become infected by a variety of bacterial agents. Bacterial infection of such wounds may result in the formation of abscesses [15]. Such abscesses may eventually form large, localized caseous masses through the accumulation of heterophil-induced fibrin deposition. Fungal infections of deep bite wounds in captive crocodylians can result in heterophilic granulomatous inflammation [14], [16] and gingivitis [14]. Despite the superficial resemblance of the tyrannosaurid mandibular lesions to abnormalities that may stem from crocodylian poxvirus, abscesses, granuloma, bite-wound gingivitis, and bite trauma, the trichomonosis-type lesions in tyrannosaurids differ in their location and distribution at the caudal end of the mandible (Figure 1 and Figure 2).

The only known disease among extant crocodylians affecting similar areas on the mandible is a skin infection caused by *Fusarium*, a filamentous fungus that is normally part of the crocodylian oral flora [14], [17]. Manifestation of this type of dermatomycosis, however, is strictly necrotic, not erosive; the infection does not lyse the underlying bone. The bone remains present but dead rather than being lysed by the infection. Also, the disease occurs only in hatchlings and relates principally to nesting conditions [14], [17].

The modern avian differential diagnostic possibilities for these tyrannosaurid mandibular lesions include vitamin C or D deficiency, aspergillosis, avian (raptor) pox, capillariasis and trichomonosis. Vitamin C or D deficiencies in extant birds and crocodylians can lead to a general decrease in bone density and integrity [14], [18] and regionalized thinning of bone [14]. This diffuse bone loss differs from the focal trichomonosis-type lesions in tyrannosaurids. The opportunistic fungal infection, aspergillosis, can lead to ‘space occupying masses in the skin’ of raptors [18], [19]. Erosive lesions form by pressure erosion from the overlying dermis, as might occur with a fibriscess. However, there are no reported occurrences of osteological lesions from aspergillosis on avian mandibles. Avian pox represents a viral disease in the Poxviridae clade known to lead to large papules in the skin and adjacent layers [18], [19]. These lesions typically occur ventral to the orbit and caudal to the jaw articulation [18]. No specific presentation is reported in the mandible. *Capillaria* is a nematode parasite that commonly forms granulomatous plaques on the palate of raptors [18], [19]. If the infestation progresses, the infection of oral mucosa can lead to ‘localized’ mandibular abscesses [18], [19]. However, these abscesses are rare, and if present, they are isolated.

Avian trichomonosis is a strongly erosive parasitic infection caused by the flagellate protozoan *Trichomonas gallinae* [20]. There are numerous strains of *T. gallinae*, some of which cause no clinical signs and possibly provide immunity against other, highly pathogenic strains [20], [21]. In a non-immune bird, infection can cause necrotic ulceration in the upper digestive tract, principally in the mouth, oesophagus, crop and protoventriculus [20]. Columbiforms are the most common host for avian trichomonosis [20], with most wild and almost all domestic pigeons being infecting with *T. gallinae* [22]. The parasite also occurs commonly in galliforms (turkeys and chickens) [7] and avivorous falconiforms (raptors) [21]–[26]. Galliforms acquire *T. gallinae* through drinking contaminated water [7], [27], while falconiforms acquire it through foraging on infected columbiform prey [21]–[26]. Vertical transmission from parents to offspring has also been demonstrated among falconiforms, either through nestlings being fed meat with adherent flagellates in it or by direct bill-to-bill contact with infected parents [21], [22], [23], [26]. The disease can have the most serious effects in young birds, often causing rapid weight loss and eventually death [21], [26].

**Figure 3:**
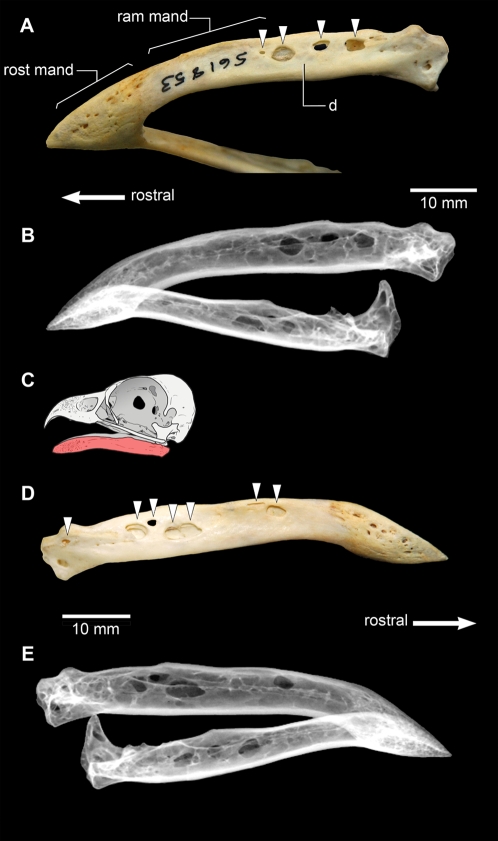
The mandible of a modern falconiform, *Pandion haliaetus*, the osprey (USNM 561853), exhibiting multiple trichomonosis lesions (indicated by arrows); the animal most likely acquired the disease by feeding on an infected pigeon [31]. (A) (photo) and (B) (radiograph), mandible in left ventrolateral view, exhibiting multiple oblate to cylindrical erosive lesions. (C), schematic interpretation of the normal skull of *Pandion haliaetus* (Queensland Museum O31935) in left lateral view with the mandible shown in red. Anatomical abbreviations: d, dentary; ram mand, mandibular ramus; rost mand, mandibular rostrum. (D) (photo) and (E) (X-ray), mandible in right lateral view, exhibiting multiple oblate to cylindrical erosive lesions. The X-rays show that the lesions are largely resolved, except for a slight radiodensity indicative of thickening along the lesion edges.

In severe cases, trichomonosis in falconiforms leads to the formation of large oropharyngeal lesions [21], including erosive deformations of the craniomandibular apparatus [26] (Figure 3). In osteological specimens that we have observed, these erosive deformations are typically cylindrical or oblate in shape, and may occur on one or both of the mandibular rami, either fully penetrating the bone, or just the lateral surface. They are randomly positioned and not symmetrical in their distribution between the rami. Significantly, they tend to concentrate in the caudal portion of the mandibular ramus, usually on the dentary, the surangular, the angular and the articular (the sutures between these bones in many falconiforms often fuse early in ontogeny [28], so that only a single mandibular element may be present). Such osteological deformations are large relative to the size of the mandible, covering much of the bone surface in certain areas (Figure 3). The margins of the lesions appear smooth, with no apparent alteration to the surrounding bone texture. In some instances, the deformations demonstrate multiple stages of development, with internal trabecular struts eroded at their centre (Figure 3, A and B).

## Discussion

The distribution, size, shape and nature of trichomonosis-type lesions in tyrannosaurid specimens are similar in nature to those manifested in modern falconiforms infected with the protozoan *Trichomonas gallinae*. Our differential diagnosis of possible diseases within the phylogenetic bracket of tyrannosaurids and modern falconiforms supports the diagnosis of a trichomonosis-type disease in these non-avian theropod dinosaurs. Thus, there is a good possibility that the disease we have identified in tyrannosaurids is homologous with modern avian trichomonosis. Indeed, the diagnosis of a disease with strong similarities to a modern transmissible avian infectious disease in tyrannosaurids suggests not only that they may have been susceptible to a similar or even the same parasite, but also that the innate immune response of some basal coelurosaurian theropods to chronic infection was similar to that which now occurs in their living descendents. Comparable lesions in modern birds are prevented from spreading throughout the medullary cavity due to the focalization of granulomatous inflammation by profound fibrin secretion elicited by the innate immune response of heterophils [29]. Therefore, it can be hypothesized that the lesions present in the tyrannosaurid specimens examined in this study are discrete, and do not invade substantially into surrounding parts of the mandible as a result of a similar innate immune mechanism. This differs from the neutrophil-induced development of extensive purulent osteomyelitis that is seen in mammals [30].

Given the ways in which *Trichomonas* infection is spread among extant birds, the occurrence of a similar disease in tyrannosaurids suggests five possible scenarios for transmission: water-borne transmission, feeding of tainted prey to nestlings, consumption of infected prey, cannibalism, and snout to snout contact during face biting between adults or between infected adults and nestlings. Water-borne transmission, although possible, cannot be demonstrated from available evidence. A causal link of individuals to one particular area with a contaminated water supply cannot be demonstrated with tyrannosaurids, although taphonomic evidence can demonstrate such a phenomenon with multiple coincident individuals [32]. The second scenario – feeding of tainted prey to nestlings [21], [22], [23], [26] – is hard to evaluate for tyrannosaurids due to a lack of available material. If infection of a *Trichomonas*-type protozoan could be acquired during the tyrannosaurid nestling stage we would expect to find occasional nestlings that had died of an acute disease course due to a naïve immune system as is seen in modern birds. It remains to be seen whether these individuals would have had the time to develop lesions before death or not because we lack tyrannosaurid nests or nestlings, so this hypothesis is hard to evaluate.

Consumption of infected prey is an important mode of *Trichomonas* transmission among modern birds of prey [21]–[26]. From traces left on bones, tyrannosaurids can be shown to have consumed a wide variety of dinosaur prey as is evidenced by bite marks on hadrosaurs, ceratopsians, other ornithischians and tyrannosaurids [33], [34]. Despite extensive records, no ornithischians of the Late Cretaceous have been reported to bear trichomonosis-like lesions. Resorptive lesions are found in the frill of some ceratopsians, as reported recently [35], but the distribution and character of these lesions are different enough to remove them from any suspicion of relationship to those found in tyrannosaurids. For one, the resorptive lesions on ceratopsian frills are very substantial in nature [35]. Secondly, the location of these lesions bear no relationship to those found in tyrannosaurids or modern birds known to have trichomonosis. Therefore, we believe that one of the ways that tyrannosaurids may have acquired the parasite was through feeding on sympatric or conspecific tyrannosaurids, in addition to other undocumented prey that may have acted as a host.

Although known to occur in basal allosauroids such as *Sinraptor dongi* [1], bite marks (deep gouges, conical, depressed fractures etc) thought to be associated with head-biting behavior are most commonly found in large-bodied tyrannosaurids. Of well-sampled collections, the relative frequency of these bite wounds is as high as 44% for mature individuals and 60% for sub-adults [1], with the implication being that head-biting behavior was characteristic of the clade. The coupling of pathological evidence for both head-biting behavior and a *Trichomonas-*type infection in tyrannosaurids may be more than just coincidental as is shown by the co-occurence of these features in the specimens that we examined [Supporting Information S1]. Head or face-biting behavior relating to intraspecific territoriality, social dominance, courtship, feeding or some other unknown aspect of tyrannosaurid behavior would have provided the ideal mechanism for the transmission of this trichomonosis-like disease.

Importantly, we do not claim that these erosive lesions are bite wounds, but instead that the evidence supports the possibility of transmission via snout-to-snout contact in a modification of the bill-to-bill transmission that can occur in living birds [21], [22], [23], [26]. Sporadic acquisition via cannibalism and feeding on unknown infected sources should not be ruled out as part of the complex ecology of this disease.

**Figure 4:**
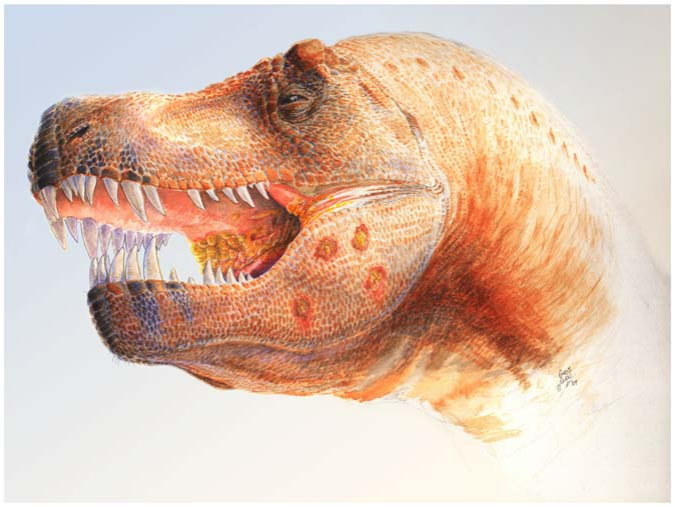
Hypothesized reconstruction of the *Trichomonas*-like infection of the oropharynx and mandible of MOR 980, commonly known as ‘Peck's Rex’ (Figure 2G). Note the yellowing of the oropharyngeal area at the back of the mouth and developed lesions within the mandible that penetrate the full thickness of the bone. This reconstruction is based on photographs of living birds and bird necropsies of individuals with trichomonosis. Illustration by Chris Glen, The University of Queensland.

Disease development of a triomononosis-type disease in tyrannosaurids likely followed the same trajectory as in modern birds. Regardless of the scenario of initial infection with a *T. gallinae*-type protozoan, an infection within the oropharynx would then have seeded protozoa into surrounding tissues by invading through the mucosal surface, as is seen in modern birds [18], [19]. Lesions represent focal sites of chronic infection within the mandible that can be reconstructed to give a general sense of the appearance of the lesions in life (Figure 4). The size and distribution of these lesions in the different individuals we observed suggest the severity of disease course in these animals.

For a trichomonosis-like disease in tyrannosaurids to progress to the point where it resulted in erosive deformation of the mandibular bones, multiple distributed oropharyngeal lesions must have been present, in addition to necrotic ulceration in the upper digestive tract, principally in the mouth and oesophagus (Figure 4). As with modern birds with trichomonosis [24], [26], feeding may have become difficult once the disease progressed to this stage, and it is very probable that *Tyrannosaurus rex* FMNH PR2081 (‘Sue’) and other more seriously affected individuals succumbed to starvation. In a current poignant example, *Sarcophilus harrisi*, the Tasmanian devil, is undergoing a similar fate today. Face-biting in devils transmits a type of malignant debilitating oral cancer, and infected individuals find themselves ultimately unable to eat [36]. Through the vehicle of disease, this head-biting behavior is bringing about a population bottleneck. In the late Maastrichtian, a disease like modern-day avian trichomonosis may have been the scourge of tyrannosaurids, thanks in part to their antagonistic behavior.

Whether or not the lesions in tyrannosaurids were caused by *Trichomonas gallinae* or a similar organism may be impossible to discover at this time, but we have provided evidence of homology of disease transmission, lesional morphology and immune response. These multiple lines of evidence within archosaur phylogeny indicate that this trichomonosis-type disease may represent the origins of modern avian trichomonosis. The record of infectious diseases of non-avian dinosaurs may yet provide us with an understanding of the development of modern avian disease and avian immunity.

## Materials and Methods

### A: Morphological examinations

Morphological measurements of mandibular skeletal elements were taken for each specimen to give general dimensional information. This was followed by measurement of observed lesion dimensions, and the location of the lesion in reference to anatomical landmarks such as the surangular foramen and articulations between elements. The subsequent description of oral lesions was conducted using descriptive terminology that took into account alterations, texture, and deformation of the surrounding bone. Radiographs of USNM 561853 (*Pandion haliaetus*) were taken on a digital radiography system at the National Museum of Natural History, Ichthyology Department at 55 kVp and 70 seconds exposure time. The subsequent differential diagnosis was undertaken with comparison to veterinary literature, texts and consultation on similar veterinary cases.

### B: Tyrannosaurid specimens examined

Sixty one tyrannosaurid specimens were examined for this study: *Albertosaurus* sp. MOR 657, *Albertosaurus* sp. RTMP 1999.50.10, *Albertosaurus* sp. RTMP 1998.68.35, *Albertosaurus* sp. RTMP 1983.29.01, *Albertosaurus* sp. 1985.98.01, *Albertosaurus* sp. RTMP 1967.9.164, *Albertosaurus* sp. RTMP 2003.45.84, *Albertosaurus* sp. RTMP 1997.50.02, *Albertosaurus* sp. RTMP 1998.63.82, *Albertosaurus* sp. RTMP 1981.3.6, *Albertosaurus* sp. NMNH 16475, *Albertosaurus* sp. RTMP 1999.50.170, *Albertosaurus* RTMP 1981.10.1, *Albertosaurus libratus* AMNH 5458, *Albertosaurus libratus* AMNH 5336, *Albertosaurus* sp. RTMP 1981.10.1, *Albertosaurus* sp. RTMP 1986.49.29, *Albertosaurus* sp. RTMP 1992.36.749, *Albertosaurus* sp. RTMP 1983.36.134, *Albertosaurus* sp. RTMP 1994.12.602, *Albertosaurus* sp. RTMP 1995.05.01, *Albertosaurus* sp. RTMP 1982.28.01, *Albertosaurus* sp. RTMP 2002.12.11, *Albertosaurus* sp. NMNH 12814, *Albertosaurus* sp. RTMP 1994.12.155, *Albertosaurus* sp. RTMP 2001.12.12, *Albertosaurus* sp. RTMP 1990.56.6, *Albertosaurus* sp. RTMP 1996.12.142 *Albertosaurus* sp. RTMP 1992.36.390, *Tyrannosaurus* (labelled *Aublysodon*) sp. RTMP 1985.62.01, *Daspletosaurus* sp. RTMP 1975.11.03, *Daspletosaurus* sp. RTMP 1994.143.1, *Daspletosaurus* sp. MOR 590, *Tyrannosaurus rex* (labelled *Nanotyrannus*) RTMP 1995.06.07, Tyrannosaurid sp. RTMP 1986.03.02, Tyrannosaurid sp. RTMP 1987.46.01, Tyrannosaurid sp. (Proprietary RTMP), Tyrannosaurid sp. RTMP 1983.29.02, Tyrannosaurid sp. 1998.93.12, Tyrannosaurid sp. RTMP 2002.12.101, Tyrannosaurid sp. RTMP 1996.03.13, Tyrannosaurid sp. RTMP 1994.12.155, Tyrannosaurid sp. RTMP 2001.12.02, Tyrannosaurid sp. MOR 1130, *Tyrannosaurus* sp. NMC 8540- cast, *Tyrannosaurus rex* CM 9380 (holotype), *Tyrannosaurus rex*, FMNH PR2081 ‘Sue’ (from original and casts on exhibit, and literature [3]), *Tyrannosaurus rex* RTMP 1992.15.1- cast, *Tyrannosaurus rex* RTMP 2001.12.147, *Tyrannosaurus rex* RTMP 81.6.1 ‘Black Beauty’, *Tyrannosaurus rex* BHI 3033 (seen as cast - ‘Stan’ at NMNH), *Tyrannosaurus rex* MOR 980 (‘Peck's Rex’), *Tyrannosaurus rex* MOR 555, *Tyrannosaurus rex* MOR 1128, *Tyrannosaurus rex* MOR 1126 *Tyrannosaurus rex* MOR 1628, *Tyrannosaurus rex* MOR 008, *Tyrannosaurus rex* MOR 1125, *Tyrannosaurus rex* AMNH 5027.

## Supporting Information

Supporting Information S1 This section includes a table with co-occurrence of face-biting evidence and the trichomonosis-type disease (Table S1), a description of archosaur mandibular morphology (Text S1), and descriptions of pathology in tyrannosaurid specimens (Text S2)
(0.07 MB DOC)
