# Supplementary material for: Common Avian Infection Plagued the Tyrant Dinosaurs
Source: PLoS One. 2009 Sep 30;4(9):e7288. doi: 10.1371/journal.pone.0007288 (PMC2748709; doi:10.1371/journal.pone.0007288)
Supplement: Supporting Information S1 — This section includes a table with co-occurrence of face-biting evidence and the trichomonosis-type disease (Table S1), a description of archosaur mandibular morphology (Text S1), and descriptions of pathology in tyrannosaurid specimens (Text S2) (0.07 MB DOC) [file pone.0007288.s001.doc]

**Supporting Information**

**Table S1**: Tyrannosaurid specimens displaying trichomonosis-type mandibular lesions. Additional information on each specimen is given in Text S2.

**that tha**

| **Specimen** | **Distribution** | **Lesion description** | **Additional evidence of face-biting** |
| --- | --- | --- | --- |
| Tyrannosaurus rex FMNH PR2081 ‘Sue’  (Figure 1 A-E) | Bilateral | Round, tapered edge, rim thickening/remodelling, infrequent median process |  |
| *Tyrannosaurus rex*  CM 9380 (holotype)  (Figure 2 A) | Unilateral | Round, tapered edge, rim remodelling | Yes |
| *Daspletosaurus* sp. RTMP 2001.36.01 (Figure 2 B-C) | Bilateral | Round to slit-shaped, tapered edges, remodelled | Yes |
| *Albertosaurus sarcophagus*  RTMP 1981.10.01  (Figure 2 D) | Unilateral | Slit-shaped, tapered edge, remodelled |  |
| *Tyrannosaurus rex*  MOR 1125 (Figure S1 E) | Unilateral | Round, tapered edge, rim remodelled |  |
| *Daspletosaurus* sp.  RTMP 1994.143.1  (Figure S1 F) | Unilateral | Slit-shaped, tapered edges, remodelled | Yes |
| *Tyrannosaurus rex­*  MOR 980 (Figure S1 G) | Unilateral | Round, to sub-oval, tapered edge, rim remodelling, minor lesion on opposite side |  |
| *Gorgosaurus* sp*.*  RTMP 2002.12.11  (not figured) | Unilateral | Round, tapered edges, remodelled |  |
| Tyrannosaurid sp. indet.  RTMP 1998.93.12  (not figured) | Unilateral | Ovoid, smooth edges, one margin thickened |  |
| *Tyrannosaurus rex*  AMNH 5029 (not figured) | Unilateral | Rounded rectangle, tapered edge, remodelled |  |

**Text S1: Archosaur mandibular morphology**

The mandibular ramus in archosaurs usually comprises at least seven bones: the dentary, the splenial, the angular, the prearticular, the surangular, the coranoid and the articular. Paired mentomandiblar ossifications also often develop within the mandibular symphysis early in ontogeny [S1,S2]. Many ornithischian dinosaurs additionally possess a predentary bone, and a supradentary ossification is known to occur in saurischians. In mesoeucrocoylians, the prearticular is lost [S3], while a coronoid is not normally present in birds.

In all archosaurs, there is typically a single external mandibular fenestra at the junction between the dentary, surangular and angular; in birds, this area forms the caudal intramandibular flexion zone. A second, more caudally positioned fenestra occurs in the mandible of some birds. Medially, there is typically one intramandibular fenestra between the splenial and the angular; a second intramandibular fenestra occurs between the coronoid and splenial in crocodylomorphs. A mylohyal foramen may also perforate the splenial close to its rostral border with the dentary.

At least two foramina perforate the external surface of the surangular in archosaurs in the area close to where some of the pathologies of interest described herein occur. Both these foramina are greatly reduced in extant birds and crocodylians. In non-avian dinosaurs, the external surangular foramina are thought to have been associated with cutaneous rami of the inferior alveolar nerve, as in modern lizards [S4]. The caudal surangular foramen is large in all tyrannosauroids [S5-7]. With the possible exception of *Acrocanthosaurus* [S8,S9], this foramen is considerably smaller in other theropods in proportion to the overall size of the mandible.

In crocodyliforms, the majority of pseudosuchians, ornithodirans (including dinosaurs) and some birds, numerous neurovascular foramina perforate the external surface of both the maxillary rostrum and the mandible. In extant crocodylians and their semi-aquatic mesoeucrocodylian relatives, these foramina are densely arranged in a ‘beehive-like’ hexagonal pattern, and correspond with ‘Dome Pressure Receptors’ (DPRs) within the dermis. These DPRs are innervated by the maxillary and mandibular rami of the trigeminal nerve [S10]. Dinosaurs, suchians, and basal crocodylomorphs ordinarily display a simpler, more linear arrangement of neurovascular foramina, dorsal and ventral to the tooth row, as in lizards [S10]. Large theropods, such as tyrannosaurids, are unusual in that there is an additional row of neurovascular foramina on the dorsal part of the maxilla, circumventing the rostral margin of the antorbital fossa [S11]. Smaller foramina also occur over the surface of the maxilla and the rostral part of the dentary, but the pattern is less dense and more random than occurs in semi-aquatic mesoeucrocodylians.

**Text S2: Tyrannosaurid specimen descriptions**

*Tyrannosaurus rex* FMNH PR 2080 ‘Sue’ (Figure 1 A-E)

The left mandible bears seven notable full thickness erosive lesions three of which occur on the dentary, three on the surangular, and one near the surangular articular margin. The right dentary, not figured, is similarly affected with multiple full thickness erosive lesions but was dorso-ventrally compressed and sheared necessitating substantial reconstructive interpretation. A very thorough description of these lesions has been published previously [S7]. The character of these lesions is very similar to those subsequently described on the holotype and MOR 980. Each lesion on the left mandible has a narrow tapered interior irregular margin with smoothed edges and a surrounding oval to sub-rectangular rim, which is in this case elevated slightly above the bone surface on the medial margin of the lesions. An exception to this pattern is the critical presence of minor projections of bone into the center of several of the caudal lesions. These small struts were previously mentioned as [4] central to the diagnosis of actinomycosis in these individuals, but are reinterpreted as being remnants of bone from the developing trichomoniasis lesions.

*Tyrannosaurus rex* CM 9380 (holotype) (Figure 2 A)

On the lateral surface of the right dentary, approximately 7.25 cm cranial to the caudal articulation of the element with the surangular along the approximate midline, there is a full thickness erosive lesion. The erosion forms a depressed oval with a surrounding rim that has a rectangular profile. The opening measures approximately 2.0cm CC, and 2.70cm DV. The outer rim measures approximately 2.90cm CC and 3.75 cm DV. Although the thinnest point of the lesion appears slightly jagged in the photograph, this is an artifact of preparation. The edges are smooth and tapered. The surrounding rim shows little evidence of remodelling except perhaps for some slight thickening and elevation along the dorsal margin of the lesion.

*Daspletosaurus* sp. RTMP 2001.36.01 (Figure 2 B and C)

Milk River, Manyberries Locality, Alberta

On the medial surface of the right pre-articular element at the caudal articulation there is a small circular erosive lesion measuring 0.46cm RC, 0.23cm ML and 0.31cm DV. The edges of the lesion are smooth and well-rounded.

There is also an ovate erosive lesion located approximately 5.4cm caudal to the rostral articulation of the left surangular element. The lesion measures 1.03cm RC and 199cm DV and has thin tapered rounded edges and a thickened surrounding rim. Associated with the caudal edge of the lesion is an approximately 7.00cm DV thickened double ridge with an 1.00cm central furrow terminating into a full thickness post-mortem fracture through the surangular. The associated bone texture is blocky within the furrow and smooth and unremodeled on either side.

On the medial surface of the right surangular element there are four full thickness ovate perforations visible ranging in size from approximately 0.50-1.20cm in diameter and one slit-like perforation 3.00cm RC and 0.20cm DV. The edges of these lesions are smooth and tapered, and the slit-like lesion appears to be two adjoining ovate lesions which have coalesced.

*Albertosaurus sarcophagus* RTMP 1981.10.01 (Figure 2 D)

Dry Island Buffalo Jump, Judith River Group, Trochu, Alberta

On the lateral surface of the left surangular element approximately 15.00 cm caudal to the rostral articulation there are two associated features. The first is a slit-shaped lesion approximately 6.00 cm rostral to the surangular fenestra that measures approximately 1.80 cm RC and 0.20 cm ML with smooth, tapered edges. The lesion is obscured by diagenetic fracturing and overlapping of surrounding fragments of the surangular. Approximately 1.20cm rostral to this first lesion and 1.00cm dorsal to it is an approximately 7.00cm RC and 4.00cm DV region of highly rugose remodeled bone with radiating folds that extend out from the region of the first lesion.

*Tyrannosaurus rex* MOR 1125 (Figure 2 E)

B-Rex Site, Hell Creek Formation, Fort Peck Lake, near Jordan, MT. 2002

On the lateral surface of the right dentary element 23.5cm rostral to the surangular element articulation and 8.25cm dorsal to the ventral edge is a circular full thickness perforative lesion measuring 1.43cm RC and 1.36cm DV. The lesion has smooth tapered edges and a surrounding region of depressed bone measuring 4.51cm RC and 3.77cm DV. There is no apparent surrounding remodeling of the bone surface.

Taphonomic interpretation: The surrounding depression of the bone surface initially appears diagnostic of some process affecting the bone surrounding the lesion; however, the surrounding diagenetic fracturing of the bone indicates a longitudinal axis of compressive stress. Stress applied to the circular lesion during burial probably caused the surrounding strain deformation of the bone surface profile, not a disease process.

*Daspletosaurus* sp. RTMP 1994.143.1 (Figure 2 F)

Quarry 215, Dinosaur Provincial Park, Alberta

There are two erosive lesions on the lateral surface of the right surangular element. The first lesion is located 3.34cm rostral to the surangular fenestra, measures approximately 1.20cm RC and 0.25cm DV and has a 45 degree rostral-dorsal orientation. The second lesion is located 6.65cm rostral to the surangular fenestra measures approximately 1.0cm RC and 0.2cm DV and has a 45 degree caudal-dorsal orientation. Both lesions exhibit smooth edges and no surrounding remodelling.

*Tyrannosaurus rex* MOR 980 (Figure 2 G)

Hell Creek Formation, Fort Peck Lake, near Jordan, MT.

Three significant erosive lesions can be viewed on the lateral surface of the left caudal mandible in this specimen. One lesion (~3.5cm CC, 3.0cm DV) is located approximately 5.25 cm cranioventrally to the surangular foramen and has a squared off oval profile with an inwardly sloping caudal margin. The edges of the lesion are tapered and bevelled. A second erosive lesion (~2.5cm CC, 3cm DV) is located approximately 14.50 cm cranial to the surangular formation and has a vertical oval profile with rounded edges. A third lesion (~ 3.0cm CC, 3.2cm DV) is located approximately 8 cm caudal to the cranial margin of the left surangular towards the dorsal surface. This lesion has a round profile and moderately sloped and tapered edges.

*Gorgosaurus* sp*.* RTMP 2002.12.11 (not figured)

Dinosaur Provincial Park, Alberta

On the lateral surface of the left dentary element approximately 2.00cm rostral to the surangular element articulation there is a resorptive lesion measuring approximately 1.50cm RC and 2.30cm DV. The lesion has smooth well-rounded edges and no evidence of surrounding remodeling.

Tyrannosaurid sp. indet. RTMP 1998.93.12 (not figured)

Dinosaur Provincial Park, Alberta

A perforative lesion 2.80cm caudal to the rostral surangular articulation measuring 1.15cm RC and 0.29cm DV. This lesion has a blocky ovate shape with smooth edges and some thickening of the bone along the rostral edge.

*Tyrannosaurus rex* AMNH 5029 (not figured)

Hell Creek Formation, Montana

On the right angular element approximately 1.50cm ventral to the dorsal articulation there is a large erosive lesion measuring approximately 0.80cm RC and 2.50cm DV with smooth tapered edges and a semi-rectangular profile. There is no significant bone remodeling surrounding this lesion.

**References**

S1. Romanoff AL (1960) The Avian Embryo. MacMillan, New York. 1305 p.

S2. Baumel JJ, Witmer LM (1993) Osteologica. In: Baumel JJ, King AS, Breazile JE, Evans HE, Vande-Berge J, editors. Handbook of Avian Anatomy: Nomina Anatomica Avium. 2nd Edition ed. Cambridge, Massachusetts: Nuttall Ornithological Society 23. pp. 45-132.

S3. Clark JM (1994) Patterns of evolution in Mesozoic crocodyliformes. In: Fraser NC, Sues H-D, editors. In the shadow of the dinosaurs. New York: Cambridge. pp. 84-97.

S4. Oelrich TM (1956) The anatomy of the head of *Ctenosaura pectinata* (Iguanidea). Miscellaneous Publications of the Museum of Zoology, University of Michigan 94: 1-122, 159 figs.

S5. Molnar RE (1991) The cranial anatomy of *Tyrannosaurus rex*. Palaeontographica (Abt A) 217: 137-176.

S6. Holtz TR (2001) The phylogeny and taxonomy of the Tyrannosauridae. In: Tanke D, Carpenter K, editors. Mesozoic Vertebrate Life. Bloomington: Indiana University Press. pp. 64-83.

S7. Brochu CA (2003) Osteology of *Tyrannosaurus rex*: insights from a nearly complete skeleton and high-resolution computed tomographic analysis of the skull. J Vertebr Paleontol 22 (Supp. to No. 4): 1-138.

S8. Harris JD (1998) A reanalysis of *Acrocanthosaurus atokensis*, its phylogenetic status, and paleobiogeographic implications, based on a new specimen from Texas. New Mexico Museum of Natural History and Science Bulletin 13: 1-75.

S9. Currie PJ, Carpenter K (2000) A new specimen of *Acrocanthosaurus atokensis* (Theropoda, Dinosauria) from the Lower Cretaceous Antlers Formation (Lower Cretaceous, Aptian) of Oklahoma, USA. Geodiversitas 22(2): 207-246.

S10. Soares D (2002) An ancient sensory organ in crocodilians. Nature 417: 241-242.

S11. Rega EA, Brochu C (2001) Paleopathology of a mature *Tyrannosaurus rex*. J Vert Paleontol 21 (Suppl. 3): 92A.
